# Symptomatic joint hypermobility is not a barrier to attendance, graduation, or satisfaction for adults participating in a multidisciplinary pain rehabilitation program

**DOI:** 10.3389/fpain.2025.1472160

**Published:** 2025-03-20

**Authors:** Lindsay G. Flegge, Emma Estrella, Elizabeth K. Harris, Adam T. Hirsh, Michael A. Bushey

**Affiliations:** ^1^ Department of Psychiatry, Indiana University School of Medicine, Indianapolis, IN, United States; ^2^ Indiana University Health, Indianapolis, IN, United States; ^3^ Department of Psychology, Indiana University Indianapolis, Indianapolis, IN, United States

**Keywords:** pain, chronic pain management, pain rehabilitation, hypermobility, hypermobility spectrum disorders, hypermobile Ehlers-Danlos syndrome, patient outcomes

## Abstract

**Introduction:**

Symptomatic joint hypermobility, as found in conditions like hypermobile Ehlers-Danlos syndrome (hEDS), presents unique challenges in pain management due to associated symptoms such as chronic pain, joint instability, and dysautonomia. Despite the high prevalence of hypermobility and associated healthcare costs, there is a lack of research on effective treatments for these patients, particularly in the context of multidisciplinary pain rehabilitation programs.

**Objective:**

This study aims to compare the baseline characteristics, attendance, graduation rates, and patient satisfaction of hypermobile and non-hypermobile adult outpatients participating in a multidisciplinary pain rehabilitation program (PRP).

**Methods:**

This retrospective cohort study analyzed clinical data from 335 patients at the Indiana University Health Pain Navigation Service between January 1, 2023, and December 31, 2023. Baseline characteristics were assessed using patient-reported outcome measures, and attendance and graduation rates were tracked. Hypermobile and non-hypermobile groups were compared with independent samples t-tests and chi-squared tests. A multiple linear regression model was used to assess the impact of hypermobility diagnosis on PRP attendance, with pertinent demographic and baseline clinical scores entered as covariates.

**Results:**

Hypermobile patients differed significantly from non-hypermobile patients in demographics, including age, gender, race, education, and employment status. Despite these differences, hypermobile patients did not differ from non-hypermobile patients in PRP attendance or graduation rates. Baseline pain, depression, and pain catastrophizing scores were lower in the hypermobile group. Exit surveys indicated similar levels of overall satisfaction with the program, though hypermobile patients were less likely to report that their needs were fully met than were non-hypermobile patients.

**Discussion:**

Despite the potential for joint hypermobility to pose a barrier to participation in multidisciplinary pain rehabilitation programs, we found no evidence that patients with a hypermobile diagnosis had less participation in an intensive outpatient pain rehabilitation program. After accounting for group differences in key demographic and clinical variables, there were no significant differences in PRP attendance between hypermobile and non-hypermobile patients. Our results are encouraging regarding the potential for multidisciplinary pain rehabilitation programs to serve the needs of these patients.

## Introduction

Hypermobile joints bend beyond the typical range of motion. Hypermobility is common and can be asymptomatic or symptomatic. Asymptomatic hypermobility occurs on its own, whereas symptomatic hypermobility occurs in the context of a specific hypermobility condition, such as hypermobile Ehlers-Danlos syndrome (hEDS) (1). Recent estimates suggest symptomatic joint hypermobility may affect nearly one in 500 people (2). Patients with hEDS and other hypermobility spectrum disorders (HSD) are conceptualized as being clinically similar with comparable symptom severity (3, 4). They often experience chronic pain, joint instability, and numerous other symptoms that impact daily functioning (5). These symptoms pose unique challenges to both patients and practitioners, owing to the lack of guidelines for managing pain in these patients (6).

There is a lack of published studies on treatments for hEDS and HSD. Because chronic pain in joint hypermobility is complex and involves nociceptive, neuropathic, and nociplastic mechanisms, single modality pain management interventions may not adequately address the unique needs of individuals with hypermobility (7, 8). The dysautonomia and subluxations that frequently accompany hEDS and HSD serve as barriers to treatment as usual (9). Furthermore, patients with hEDS report multiple barriers to pain care including lack of personalized treatment, knowledge deficits about hEDS and hypermobility among clinicians, delayed diagnosis, and poor communication from care teams (10, 11). Patients with hypermobility often perceive poor health care due to high cost, distance and time required for treatment, wait times for diagnosis, and negative attitudes from healthcare providers (10, 11). Due to this myriad of factors, specialized treatment approaches are needed.

One such specialized approach, multidisciplinary pain rehabilitation, has demonstrated efficacy in chronic pain management (12). Multidisciplinary pain rehabilitation emphasizes a holistic approach, combining medical, physical therapy, and psychosocial interventions (13). The combined and collaborative nature of these programs results in improved pain outcomes and decreased disability (14, 15). These programs have also been associated with improvements in concurrent problems prevalent among patients with chronic pain, such as depression (16), insomnia (17), and work absence (18). As such, these programs offer promise for patients with hypermobility who have physical, psychological, and social needs that are not well-served by traditional pain care. Despite this, very little is known about how patients with hypermobility diagnoses engage with multidisciplinary pain rehabilitation.

Of the prior research that does exist, results are mixed and evidence is weak (19, 20). In a review of 10 studies examining exercise and rehabilitation programs for individuals with Ehlers-Danlos syndrome and HSD, results indicated improvements in physical and psychological well-being following participation (20). However, results were interpreted with caution due to moderate to critical risk of bias and mixed quality of studies. In a scoping review of 10 studies examining psychological interventions for individuals of any age with hEDS, most of the included studies reported one or more significant improvements in domains such as pain, anxiety, depression, and quality of life (21). However, results in this review also were interpreted with caution due to a lack of quality evidence and other limitations of the included studies, such as poor description of the psychological intervention. Additional studies have demonstrated maintained improvements in balance, fatigue, and quality of life for patients with joint hypermobility after undergoing multidisciplinary pain rehabilitation; however, sample sizes are very small, limiting strong conclusions and generalizations (22, 23). Moreover, we are not aware of any studies in the United States examining multidisciplinary pain rehabilitation for adults with joint hypermobility.

Over the past three years, we have developed and implemented a multidisciplinary pain rehabilitation program (PRP) in an urban outpatient hospital setting in the Midwest. A higher-than-expected volume of patients with hypermobility diagnoses have presented to our program, largely due to referrals from an affiliated genetics specialty clinic. Patients with hEDS and HSD reported a lack of program individualization for these conditions, echoing what has been previously reported by Bovet and colleagues (10). These patient reports motivated us to examine program attendance and graduation among this subgroup.

### Specific study aims

1. Compare baseline characteristics for hypermobile and non-hypermobile adult outpatients presenting for multidisciplinary pain rehabilitation.
2. Contrast attendance, graduation rates, and patient satisfaction for hypermobile and non-hypermobile adult outpatients participating in a multidisciplinary pain rehabilitation program.

## Methods

### Sample, data sources, and variables

This research was approved by the Indiana University Internal Review Board. This is a retrospective cohort study including clinical data from 335 patients seen at the Indiana University Health (IUH) Pain Navigation Service (PNS) from January 1, 2023 to December 31, 2023. The PNS is a multidisciplinary assessment clinic designed to provide patients with evaluations from a medical provider (nurse practitioner or physician with pain expertise), mental health clinician, and physical therapist. The clinic performs a diagnostic assessment and evaluates patient appropriateness for a variety of pain services, including the IUH Pain Rehabilitation Program (PRP). The PRP is a multidisciplinary three-week intensive outpatient program that incorporates pain psychology, physical therapy (PT), occupational therapy, massage therapy, yoga therapy, music therapy, nutrition, social work, chaplaincy, and peer support into 5 days per week of full-day programming. Pain psychology content is rooted in Cognitive Behavioral Therapy (CBT) and delivered in both group and individual formats. Skilled physical therapy is taught from a Pain Neuroscience Education (PNE) perspective, combines both education and physical exercises, and is delivered 3×/week. Each week, providers from these disciplines hold a one-hour virtual meeting to discuss patient progress, with each discipline present providing updates on each patient. Comments from each specialty are collected in a rounding report. Specialties unable to attend the meeting can proactively enter comments, which are read during the meeting. While these meetings provide opportunities to coordinate and personalize care, there are no explicit program modifications made for hypermobility (or any other condition). A trained nurse screens referrals to the PNS to determine appropriateness. The criteria for appropriateness include that pain is chronic (has been present for ≥6 months) and that the referral is not specifically for ongoing long-term opioid therapy.

Derived variables for this analysis included age at PNS medical appointment (PNS date – DOB), distance from clinic (calculated by measuring the driving distance between patient and clinic zip code using Google Maps), and binary variables on whether patients attended any of the three PNS appointments and whether they enrolled in PRP (i.e., went to at least one day of programming). To define the hypermobile group, we used multiple ICD-10 codes that commonly apply to our hypermobile population. Diagnostic reports were run in the electronic health record searching for the ICD-10 codes Q79.6 (EDS), Q79.9 (congenital malformation of connective tissue) and M35.7 (hypermobility syndrome). Patients with any of these diagnoses documented in their chart were included in the “hypermobile” group. For a breakdown of the frequency of patients having each code or combination of codes, refer to Supplementary Table S1. All other patients were included in the non-hypermobile comparison group.

Patients completed baseline questionnaires at both their PNS medical and mental health appointments directly into the Research Electronic Data Capture (REDCap) (24, 25) web portal or on paper and coded into REDCap by clinic staff. Measures appearing on both questionnaires (described in more detail below) included: demographics, Pain, Enjoyment of Life, and General Activity (PEG) Scale (26), Patient Health Questionnaire-9 (PHQ-2/9) (27, 28), and Generalized Anxiety Disorder 7 (GAD-2/7) (29, 30). If a measure was completed at more than one appointment, the version from their medical appointment was used. Measures only administered at the medical visit included: Fibromyalgia Diagnostic Questionnaire (31), Patient-Reported Outcomes Measurement Information System (PROMIS) Physical Function 12a (PROMIS-PF) (32), and Opioid Use Disorder (OUD) symptom checklist (33). Measures administered only at the mental health visit included: the Pain Catastrophizing Scale (PCS) (34), PROMIS Social Roles & Abilities (PROMIS-SF) scale (35), Insomnia Severity Index (ISI) (36), and Primary Care PTSD screen for DSM-5 (PC-PTSD-5) (37).

For this study, data capture was completed 6/1/2024, allowing a minimum of 150 days (for patients evaluated 12/31/2023) and up to 515 days (for patients evaluated 1/1/2023) for patients to have enrolled in PRP. The mean lag from PNS medical appointment to PRP enrollment for 117 patients enrolled in PRP since 2021 was 103 days, with a range 11–727 days. At 515 days, 98% of historical patients would have been enrolled, and at 150 days, 80% would have been enrolled.

### Patient-reported measures

Multiple choice questions assessed gender, birth sex, sexual preference, race, ethnicity, highest education attainment, marital status, primary employment category, income sufficiency, and pain duration. Four additional questions asked patients to estimate the approximate amount of time they spend each week working for pay, working around the home (e.g., laundry, childcare), at school or doing coursework, or volunteering. Response options for these four questions included: None, 1–10 h, 11–20 h, 21–40 h, and >40 h.

The PEG scale is a 3-item patient-reported outcome measure that assesses pain severity (P), and the impact of pain on enjoyment of life (E) and general activity (G). Each item is rated on a scale from 0 to 10, with the total score being the average of the three items (0–10). Higher scores indicate a greater burden of pain.

The Patient Health Questionnaire-9 (PHQ-9) is a 9-item measure designed to assess the severity of depressive symptoms over the past 2 weeks based on the Diagnostic and Statistical Manual of Mental Disorders (DSM-IV) criteria. Each item is rated on a scale from 0 to 3, resulting in total scores ranging from 0 to 27. Higher scores indicate more severe depression. The PHQ-2, a screening tool, uses the first two questions of the PHQ-9. Our questionnaire employs branched logic, providing the additional seven questions to patients who score ≥2 on the PHQ-2.

The Generalized Anxiety Disorder 7 (GAD-7) is a 7-item measure used to assess the severity of anxiety symptoms over the past two weeks. Each item is rated on a scale from 0 to 3, resulting in total scores ranging from 0 to 21. Higher scores indicate more severe anxiety. The GAD-2, a screening tool, uses the first two questions of the GAD-7. Our questionnaire employs branched logic, presenting the additional five questions to patients who score ≥2 on the GAD-2.

The Fibromyalgia Diagnostic Questionnaire consists of three parts: the Widespread Pain Index (WPI), the Symptom Severity Score (SSS), and an item inquiring whether symptoms have been present for 3 months or more. The WPI asks patients to identify areas of pain from a list of 19 regions over the past week, with scores ranging from 0 to 19. The SSS is divided into two parts. Part A contains three multi-response items measuring fatigue, sleep quality, and cognitive function, with scores ranging from 0 to 9. Part B asks patients to check symptoms experienced over the past week from a list of 41 items, with scores ranging from 0 to 3 (0 = no symptoms, 1 = 1–10, 2 = 11–24, 3 = 25 or more symptoms). The total SSS score is the sum of Part A and Part B. Diagnostic criteria for fibromyalgia are met if symptoms have been present for at least 3 months and either (WPI ≥7 and SSS ≥5) or (WPI = 3–6 and SSS ≥9).

The Opioid Use Disorder (OUD) Symptoms Checklist is an 11-item measure that asks patients to report on the 11 DSM-5 diagnostic criteria for OUD. A screening question preceding this measure inquires if patients have taken any opioids in the past year, and only those who answer yes are given the 11 items. An additional item asks if patients are currently prescribed opioids, as the items addressing tolerance and withdrawal are not counted towards the final score in such patients. Scores range from 0 to 11 (0–9 for patients prescribed opioids), with scores of 2 or more indicating a diagnosis of mild OUD, and higher scores representing greater OUD severity.

The Patient-Reported Outcomes Measurement Information System (PROMIS) Physical Function 12a (PROMIS-PF) assesses physical function. An introductory question determines whether the patient can walk 25 feet on a level surface, which dictates if all 12 items or only six items are asked. Each item is scored from 1 to 5, with total scores ranging from 6 to 60. Higher scores indicate a higher level of physical function. For this study, raw scores were used.

The PROMIS Ability to Participate in Social Roles and Activities scale (PROMIS-SF) is an 8-item measure that assesses an individual's perceived limitations and abilities in fulfilling social roles. Each item is scored from 1–5, with total scores ranging from 8 to 40. Higher scores represent a greater ability to participate in social roles and activities. Raw scores were used for this study.

The Insomnia Severity Index (ISI) is a 7-item self-report measure designed to assess the nature, severity, and impact of insomnia symptoms on daily functioning. Each item is rated on a scale from 0 to 4, with total scores ranging from 0 to 28. Higher scores indicate more severe insomnia.

The Pain Catastrophizing Scale (PCS) is a 13-item self-report questionnaire that assesses an individual's tendency to engage in pain catastrophizing. Each item is rated on a scale from 0 to 4, with total scores ranging from 0 to 52. Higher scores indicate more severe pain catastrophizing.

The Primary Care PTSD Screen for DSM-5 (PC-PTSD-5) begins with a preliminary question asking if patients have ever experienced a traumatic event, with examples provided. Those who answer “yes” are given five additional yes/no questions about different PTSD symptoms experienced in the past month. Scores range from 0 to 5, with higher scores indicating more PTSD symptoms.

### Patient attendance and graduation

Patient attendance in PRP was tracked through calculating the percentage of pain psychology groups attended throughout the program. Depending on which cohort patients attended, they would have been registered for 30–33 group psychotherapy sessions. Patients are permitted to miss up to 20% of scheduled groups for any reason (e.g., sickness, lack of transportation, medical appointment conflicts) and still graduate PRP. Tracking how many groups patients are registered for compared to how many they attend is a routine part of tracking PRP attendance and electronically updated each cohort.

Furthermore, graduation rates are tracked through a simple YES/NO at the end of PRP. While it is the expectation for all patients to graduate PRP, patients may choose to leave the program at any time or may have an attendance or behavioral issue that requires early dismissal from the program.

### Patient-reported exit surveys

On the last day of each PRP cohort, patients are asked to complete an exit survey asking about program satisfaction (six 5-point Likert-response questions), perceived program difficulty (one 5-point Likert-response question), and one question asking whether their overall needs were met (yes/no/unsure response options). Patients are also asked who had referred them to the program. Patients have the option to complete the exit surveys anonymously.

### Statistical analysis

Descriptive analyses included frequencies, means, and standard deviations. For continuous variables, unadjusted between group differences were examined using independent samples Student's *T*-tests. For categorical variables, chi-squared tests were used to examine unadjusted between group differences. Where possible, categorical groups containing five or fewer patients were combined with other groups before analysis. Additionally, a multiple regression model was generated to determine the relationship between PRP completion rates and hypermobility diagnosis, adjusting for relevant demographic variables and baseline clinical measure scores. For this exploratory analysis, a *P*-value ≤.05 was considered statistically significant, though results should be interpreted cautiously given multiple comparisons. Symptom measures with unexpected missing values were excluded from analysis. “Expected” missingness occurred when branch logic caused items to be skipped for certain measures (e.g., GAD-2/7, PHQ-2/9, PROMIS-PF). The predominant reasons for “unexpected” missing values were clinic logistical issues (e.g., not having a systematic approach to ensuring measures are completed) and technical errors (e.g., a patient accidentally skipping a question). These factors were considered missing-at-random. Analyses were conducted in IBM SPSS Statistics for Windows, Version 29.0.

## Results

### Patient variables

Of the 335 patients seen in the PNS clinic in 2023, 60 had a hypermobility diagnosis and 275 did not. Between group comparisons of continuous variables are shown in Table 1, and between group comparisons of categorical variables are shown in Table 2. There were several significant group differences in patient demographic variables (Table 2). Hypermobile patients were more likely to carry commercial insurance compared to non-hypermobile patients (68% vs. 30%, *P* < .001). Regarding demographics, the hypermobile group was younger on average than the non-hypermobile group [36 (11.9) years vs. 50 (14.7) years, *P* < .001]. The hypermobile group was also less racially diverse (0% Black vs. 19% Black, *P* = .02), more likely to be female gender (86% vs. 72%, *P* = .03) and female birth sex (94% vs. 74%, *P* = .01), more likely to have a sexual preference other than straight (31% vs. 13%, *P* = .01), more likely to have higher education (37% graduate degree vs. 9% graduate degree, *P* < .001), and more likely to be employed full time (54% vs. 19%, *P* < .001).

**Table 1:** Comparison of continuous variables between hypermobile and non-hypermobile patients.

Variable	Total		Hypermobile		Non-hypermobile		*P*-value
	*N*	Mean (SD)	*N*	Mean (SD)	*N*	Mean (SD)	
Baseline							
Age (years)	335	47.5 (15.2)	60	36 (11.9)	275	50 (14.7)	<.001
Distance (miles)	334	30 (37.1)	59	50 (54.2)	275	25.7 (30.8)	<.001
Pain (PEG)	212	7.0 (1.9)	51	6.3 (1.8)	161	7.2 (1.9)	0.003
Depression (PHQ)	221	9.5 (7.7)	51	7.5 (7.8)	170	10.1 (7.6)	0.04
Anxiety (GAD)	211	7.5 (6.3)	52	6.5 (6.2)	159	7.8 (6.4)	0.21
Catastrophizing (PCS)	134	24.1 (12.9)	33	19.1 (12.9)	101	25.7 (12.5)	0.009
Physical function (PROMIS-PF)	158	20 (11)	38	21.9 (10.1)	120	19.5 (11.2)	0.23
Social function (PROMIS-SF)	136	17 (7.4)	33	19 (6.5)	103	16.3 (7.5)	0.07
Widespread pain index (WPI)	335	3.3 (5.1)	60	5.4 (6.2)	275	2.9 (4.7)	<.001
WPI # of regions	335	1.4 (2)	60	2.3 (2.3)	275	1.2 (1.9)	<.001
SSS	167	6.8 (2.6)	43	7.3 (2.2)	124	6.6 (2.7)	0.16
Insomnia (ISI)	124	14.8 (7.3)	30	12.8 (6.5)	94	15.4 (7.5)	0.09
PTSD (PC-PTSD-5)	132	2.2 (2)	32	2.7 (2)	100	2 (2)	0.12
OUD Sx checklist	95	0.5 (0.9)	18	0.1 (0.3)	77	0.6 (1)	0.04
PRP attendance rate	48	0.9 (0.3)	9	0.8 (0.2)	39	0.9 (0.3)	0.84

*P*-values were calculated using Student's *T*-test for independent samples.

PRP, pain rehabilitation program; SD, standard deviation; PEG, 3-item (pain, enjoyment, general activity) scale; PHQ, patient health questionnaire depression measure; GAD, generalized anxiety disorder measure; PCS, pain catastrophizing scale; PROMIS, patient-reported outcomes measurement information system; PF, physical function; SF, social function; PC-PTSD-5, primary care post-traumatic stress disorder screen for DSM-5; OUD Sx, opioid use disorder symptom.

**Table 2:** Comparison of categorical variables between hypermobile and non-hypermobile patients.

Variable	Total	Hypermobile	Non-hypermobile	*P*-value
	*N*	*N* (%)	*N* (%)	
Payer				<.001
Commercial	124	41 (68%)	83 (30%)	
Medicaid	98	11 (18%)	87 (32%)	
Medicare	104	7 (12%)	97 (35%)	
Other	9	1 (2%)	8 (3%)	
Referring department				<.001
Neurology	70	3 (5%)	67 (24%)	
Primary care	62	4 (7%)	58 (21%)	
PM&R or PT	46	5 (8%)	41 (15%)	
Medical genetics	42	39 (65%)	3 (1%)	
Pain management	29	2 (3%)	27 (10%)	
Rheumatology	29	3 (5%)	26 (9%)	
Psychiatry	25	1 (2%)	24 (9%)	
Medical miscellaneous	18	0 (0%)	18 (7%)	
Other	14	3 (5%)	11 (4%)	
Mental health visit				.24
No	140	21 (35%)	119 (43%)	
Yes	195	39 (65%)	156 (57%)	
PT visit				.14
No	152	22 (37%)	130 (47%)	
Yes	183	38 (63%)	145 (53%)	
All 3 visits				.54
No	196	33 (55%)	163 (59%)	
Yes	139	27 (45%)	112 (41%)	
PRP enrollment				.87
No	287	51 (85%)	236 (86%)	
Yes	48	9 (15%)	39 (14%)	
PRP graduation				.87
No	8	2 (22%)	6 (15%)	
Yes	40	7 (78%)	33 (85%)	
Race				.02
Black	24	0 (0%)	24 (19%)	
White	122	30 (88%)	92 (74%)	
Other	13	4 (12%)	9 (7%)	
Ethnicity				.87
Non-Hispanic	158	34 (97%)	124 (98%)	
Hispanic	4	1 (3%)	3 (2%)	
Gender				.03
Female	127	31 (86%)	96 (72%)	
Male	34	2 (6%)	32 (24%)	
Other	8	3 (8%)	5 (4%)	
Sex				.01
Female	125	33 (94%)	92 (74%)	
Male	32	1 (3%)	31 (25%)	
Other	2	1 (3%)	1 (1%)	
Sexual preference				.01
Straight	134	24 (69%)	110 (87%)	
Other	28	11 (31%)	17 (13%)	
Education				<.001
<High school	10	0 (0%)	10 (8%)	
High school degree	27	3 (9%)	24 (19%)	
Some college	40	6 (17%)	34 (27%)	
Associate degree	26	4 (11%)	22 (17%)	
Bachelor's degree	36	9 (26%)	27 (21%)	
Graduate degree	24	13 (37%)	11 (9%)	
Marital status				.06
Single	48	14 (41%)	34 (27%)	
Married	75	17 (50%)	58 (46%)	
Previously married	37	3 (9%)	34 (27%)	
Employment				<.001
Full time	43	19 (54%)	24 (19%)	
Part time	16	2 (6%)	14 (11%)	
Retired	24	0 (0%)	24 (19%)	
Homemaker	7	2 (6%)	5 (4%)	
Student	6	2 (6%)	4 (3%)	
STD or LTD	14	5 (14%)	9 (7%)	
SSDI	35	3 (9%)	32 (25%)	
Unemployed	18	2 (6%)	16 (13%)	
Work - pay				.009
None	88	13 (37%)	75 (63%)	
1–10 h	7	0 (0%)	7 (6%)	
11–20 h	6	1 (3%)	5 (4%)	
21–40 h	27	10 (29%)	17 (14%)	
>40 h	88	11 (31%)	16 (13%)	
Work - home				.88
None	13	2 (6%)	11 (9%)	
1–10 h	96	22 (65%)	74 (61%)	
11–20 h	32	8 (24%)	24 (20%)	
21–40 h	7	1 (3%)	6 (5%)	
>40 h	8	1 (3%)	7 (6%)	
Work - school				.54
None	131	31 (89%)	100 (88%)	
1–10 h	9	2 (6%)	7 (6%)	
11–20 h	4	2 (6%)	2 (2%)	
21–40 h	4	0 (0%)	4 (4%)	
>40 h	1	0 (0%)	1 (1%)	
Work - volunteering				.07
None	117	23 (66%)	94 (78%)	
1–10 h	31	12 (34%)	19 (16%)	
11–20 h	5	0 (0%)	5 (4%)	
21–40 h	2	0 (0%)	2 (2%)	
>40 h	0	0 (0%)	0 (0%)	
Income				.13
Comfortable	66	15 (44%)	51 (40%)	
Just enough	55	15 (44%)	40 (32%)	
NOT enough	39	4 (12%)	35 (28%)	
Pain duration				.12
<1 years	7	0 (0%)	7 (6%)	
1–3 years	15	1 (3%)	14 (11%)	
3–5 years	14	4 (11%)	10 (8%)	
5–10 years	40	6 (17%)	34 (27%)	
>10 years	86	24 (69%)	62 (49%)	
Fibromyalgia criteria met				.21
No	99	22 (51%)	77 (62%)	
Yes	68	21 (49%)	47 (38%)	

*P*-values were calculated using Chi-Squared test.

PRP, pain rehabilitation program; PM&R, physical medicine & rehabilitation; PT, physical therapy; STD, short-term disability; LTD, long-term disability; SSDI, social security disability income.

Regarding symptom measures, the hypermobile group had significantly lower pain [PEG score 6.3 (1.8) vs. 7.2 (1.9), *P* = .003], depression [PHQ-2/9 score 7.5 (7.8) vs. 10.1 (7.6), *P* = .04], and pain catastrophizing [PCS score 19.1 (12.9) vs. 24.1 (12.9), *P* = .009]. Hypermobile patients also had more widespread pain [WPI score 5.4 (6.2) vs. 3.3 (5.1), *P* < .001] and number of pain regions [2.3 (2.3) vs. 1.4 (2.0), *P* < .001].

### Health services variables

The difference in referring department for hypermobile vs. non-hypermobile patients was significant (*P* < .001), with most hypermobile patients being referred by the medical genetics department, which includes an EDS specialty clinic. In our overall sample, 182 patients (54%) had a PT visit, 195 (58%) had a mental health visit, and 139 (41%) had all three (i.e., medical, PT, mental health) visits. There were no statistically significant differences between hypermobile and non-hypermobile patients in attendance rates for these visits, even though the hypermobile group lived further from the clinic on average compared to the non-hypermobile group [50.0 (54.2) miles vs. 25.7 (30.8) miles, *P* < .001]. As with PNS visit attendance, hypermobile patients did not significantly differ from non-hypermobile patients on PRP enrollment (15% vs. 14%, *P* = .87), PRP attendance [80% (20%) vs. 90% (30%), *P* = .84], or PRP graduation rate (78% vs. 85%, *P* = .87), though these results are tentative given the uneven cell counts of the hypermobile and non-hypermobile groups.

### Multiple regression model analysis

We conducted a hierarchical multiple regression analysis to examine the relationship between hypermobility status (hypermobile vs. non-hypermobile) and PRP attendance (Table 3). In step one of the model, we entered the demographic variables of age, gender, race, education, employment, and distance to clinic. Collectively, these variables accounted for 26.7% of the variance in PRP attendance [F(6,13) = 0.782, *P* = .599]. However, neither the overall model nor any individual demographic variable was statistically significant in this step. In step two, we entered the baseline clinical variables of pain (PEG), catastrophizing (PCS), and depressive symptoms (PHQ). These variables accounted for an additional 39.2% of the variance in PRP attendance [F(9,10) = 2.132, *P* = .127], but again, neither the overall model nor the individual variables were significant. Hypermobility status was entered in step three, accounting for an additional, though non-significant, 6.9% of the variance in PRP attendance [F(10,9) = 2.395, *P* = .102]. In the final model that included all variables, baseline PCS score was the only significant predictor of PRP attendance (Beta = −0.975, *t* = −2.664, *P* = .026), although distance to clinic was just above the threshold for significance (Beta = 0.549, *t* = 2.248, *P* = 0.051).

**Table 3:** Multiple linear regression model assessing relationship between hypermobility and covariates with PRP attendance.

Variable	B	SE	Beta	*t*	*p*
Step 1: Demographic variables					
Intercept	0.890	0.360	–	2.474	0.028
Age	−0.007	0.005	−0.412	−1.397	0.186
Distance	0.001	0.001	0.175	0.628	0.541
Gender	0.007	0.108	0.017	0.063	0.951
Race	0.093	0.111	0.256	0.840	0.416
Education	0.017	0.053	0.109	0.326	0.750
Employment	−0.016	0.036	−0.132	−0.428	0.676
Step 2: Demographic variables + baseline measures					
Intercept	1.542	0.414	–	3.725	0.004
Age	−0.009	0.006	−0.531	−1.535	0.156
Distance	0.002	0.001	0.434	1.760	0.109
Gender	−0.158	0.120	−0.394	−1.318	0.217
Race	−0.024	0.094	−0.066	−0.254	0.805
Education	0.018	0.066	0.113	0.273	0.791
Employment	0.010	0.031	0.083	0.311	0.762
Pain (PEG)	0.019	0.016	0.300	1.226	0.248
Catastrophizing (PCS)	−0.020	0.010	−0.742	−2.103	0.062
Depression (PHQ)	−0.007	0.013	−0.206	−0.551	0.594
Step 3: Demographic variables + baseline measures + hypermobility					
Intercept	2.022	0.503	–	4.024	0.003
Age	−0.011	0.006	−0.623	−1.881	0.093
Distance	0.003	0.001	0.549	2.248	0.051
Gender	−0.217	0.119	−0.539	−1.814	0.103
Race	0.019	0.093	0.053	0.208	0.840
Education	0.029	0.062	0.182	0.464	0.654
Employment	−0.003	0.031	−0.027	−0.103	0.920
Pain (PEG)	0.006	0.018	0.087	0.322	0.755
Catastrophizing (PCS)	−0.027	0.010	−0.975	−2.664	0.026
Depression (PHQ)	−0.001	0.013	−0.027	−0.073	0.943
Hypermobility	−0.383	0.253	−0.427	−1.512	0.165

### Exit survey

Many patients completed the exit surveys anonymously, which made it difficult to directly compare hypermobile vs. non-hypermobile patients' responses. However, because 93% of patients referred by Medical Genetics in our sample had a hypermobile diagnosis – compared to only 7% from all other sources – we used referral by genetics as a proxy for hypermobility for this analysis. Overall, program satisfaction was roughly equivalent between patients referred by genetics and other patients [4.4 (0.7) vs. 4.7 (0.4), *P* = .11], though genetics referrals were less satisfied with the program organization compared to other patients [3.9 (0.8) vs. 4.6 (0.6), *P* = .01; Table 4]. Although patients rated the program as moderately difficult [overall mean (SD) 3.6 (0.9) out of 5], this rating did not differ based on referral source. Despite the similarities in satisfaction and perceived difficulty scores, genetics referrals were less likely to report that the program met their needs compared to other patients (57% vs. 74% indicated “Yes” that their needs were met, *P* = .03).

**Table 4:** Exit survey responses - genetics referrals vs. other patients.

Survey item	Genetics referrals^a^ (*N* = 7): Mean (SD)	Other patients (*N* = 26): Mean (SD)	*P*-value
Satisfaction scores^b^			
A. Program organization	3.9 (0.79)	4.6 (0.6)	.01
B. Instructor's knowledge about pain management	4.6 (0.8)	4.9 (0.4)	.19
C. Topics presented during the program	4.9 (0.4)	4.7 (0.9)	.64
D. Facility rooms and space^c^	4.0 (1.5)	4.5 (0.8)	.26
E. Registration and administration	4.6 (1.1)	4.9 (0.3)	.21
F. Value of the program	4.6 (0.8)	4.9 (0.4)	.19
Average Satisfaction	4.4 (0.7)	4.7 (0.4)	.11
Perceived difficulty^d^	*n* = 6	*n* = 23	
Scores	3.8 (0.9)	3.5 (0.9)	.50
Overall needs met	*N* (%)	*N* (%)	
Yes	4 (57%)	17 (74%)	.03
No	2 (29%)	0 (0%)	
Unsure	1 (14%)	6 (26%)	

^a^ Genetics Referrals are highly likely to have a hypermobile diagnosis.

^b^ Satisfaction Scores ranged from 1 (very unsatisfied) to 5 (very satisfied).

^c^ 1 “other” patient left the Facility field blank (*n* = 25 other patients for this item).

^d^ Perceived Difficulty scores ranged from 1 (extremely easy) to 5 (extremely hard).

## Discussion

Our study had several noteworthy findings. First, hypermobile patients differed from non-hypermobile patients on many demographic characteristics, including age, sex, race, education, employment, pain duration, and insurance payer. Second, hypermobile patients had lower baseline pain, depression, and catastrophizing scores on average than non-hypermobile patients. Third, hypermobile patients did not differ from non-hypermobile patients in PRP attendance or graduation rates, despite living further from the clinic on average. Fourth, patients likely to be hypermobile (i.e., referred by genetics) were less likely to report that the program met their needs, despite reporting similar satisfaction with the program and similar difficulty.

Certain demographic factors may serve as facilitators of PRP participation. The finding that many baseline demographic characteristics were significantly different between hypermobile and non-hypermobile patients is not surprising. Multiple studies have demonstrated that hEDS and other hypermobility conditions are more likely to be diagnosed in younger White females with higher education (38). Being educated and gainfully employed may make it easier for hypermobile patients to participate in PRP, as these demographic characteristics are commonly conceptualized as facilitators of positive health outcomes (39, 40). Similarly, the social advantages of being White may also translate into more accessible PRP participation for hypermobile patients (41). Also, the fact that, at baseline, our hypermobile patients presenting for initial evaluations lived further from the PRP could potentially be a factor in participation. On one hand, one might expect that living farther away would be a barrier to program participation. On the other hand, it may be that a willingness to commute from farther away selects highly motivated patients who are more likely to participate and graduate from PRP. This reasoning is consistent with previous research on pain self-efficacy mediating interdisciplinary pain rehabilitation program outcomes (42).

Additionally, our hypermobile sample had lower pain catastrophizing scores and lower pain intensity and depression scores at baseline, which could also contribute to a greater ability and willingness to participate in more intensive pain treatment programming. It is important to note that pain catastrophizing was associated with lower PRP attendance in our sample. This is concerning given that patients who engage in high catastrophizing tend to have worse pain, mood, and adaption to chronic pain and, thus, may be in greater need of intensive pain care (43).

Although the potential facilitating factors (e.g., employment status, education) may have offset the potential barriers posed by hypermobility, our results corroborate recently published findings from a study utilizing the Swedish national registry. That study reported data from multiple multidisciplinary pain rehabilitation programs in Sweden over 8 years and assessed differences from pre- to 1-year follow-up after completion. They found no differences in treatment outcomes between hypermobile and non-hypermobile patients (8). Our results affirm the Swedish findings and, collectively, suggest that patients with hypermobility can participate in multidisciplinary pain rehabilitation without major changes to standard programming.

The findings that hypermobile patients did not differ from non-hypermobile patients in PRP attendance or graduation rates are novel, as we are unaware of any previous studies that have examined these outcomes. Previous studies have reported unique considerations for hypermobile patients, such as increased risk of joint subluxations or iatrogenic injuries that contribute to dissatisfaction with care (10). These challenges may then lead to expectations that hypermobile patients are less able to participate in pain rehabilitation. By contrast, our findings highlight the potential for PRPs to be similarly accessible for both hypermobile and non-hypermobile patients and suggest that patients with hypermobility can successfully engage in intensive rehabilitation programs, possibly even without specific modifications or custom support. This is crucial as it broadens the scope of potential beneficiaries of multidisciplinary pain rehabilitation and underscores the importance of including hypermobile patients in these programs without bias or preconceived notions.

Our exit survey results must be interpreted with caution due to the small sample size. Further, because we can only surmise whether respondents were hypermobile based on the frequency of diagnoses from different referral sources, we cannot be certain that we have a clean separation of hypermobile vs. non-hypermobile patients. With those caveats, it is noteworthy that, despite overall strong satisfaction scores with the program and an average perceived difficulty level (3.6 out of 5) that is consistent with a description of “challenging but not overwhelming”, patients referred by genetics (and likely hypermobile) were less likely to report that the program met their overall needs. These results are consistent with existing literature that demonstrates many patients with hypermobility report dissatisfaction with healthcare due to lack of understanding of their condition among medical professionals (12). Our current exit survey was not designed to probe further into what specific needs were not met; however, it would be worthwhile to add such inquiry to future exit surveys or add post-PRP qualitative interviews to see if a needs gap might be identified for these patients.

### Strengths and limitations

This study addresses a gap in the literature regarding how patients with hypermobility diagnoses present at baseline and engage with multidisciplinary pain rehabilitation programming. A strength of the study is its use of data collected in real-world clinical care, which improves its generalizability compared to more controlled studies. However, this is also a limitation, as this approach may have contributed to missingness in data (i.e., without the ability to provide financial incentives for patients to complete measures). Further, analysis is limited to the data that have been collected, so certain variables of interest – such as the frequency of subluxation or other similar outcomes in our patients – were not available. These data limitations also produced uneven cell counts between the hypermobile and non-hypermobile groups. Likewise, our reliance on diagnostic codes typically associated with hypermobility is pragmatic but not ideal. It is also possible that our rehabilitation program, specifically our physical therapists, may have unknowingly adapted our approach to accommodate the high volume of patients with a hypermobility diagnosis. There is also a potential sampling bias, as most patients with hypermobility identified as women, with a majority being White, and the patients had a limited age range; however, these demographics are consistent with previous research (36). Furthermore, given our hypermobile patients were more likely to live further from the clinic, we cannot rule out socioeconomic disparities limiting participation from underprivileged groups in our sample. Lastly, the exit surveys, while informative, were partially anonymous and insufficient in number for meaningful quantitative analysis.

### Clinical implications

This study underscores the potential for patients with hypermobility to successfully participate in multidisciplinary pain rehabilitation. Despite expected challenges traditionally associated with hypermobility, including joint instability, dysautonomia, and frequent subluxations, patients demonstrated comparable attendance and graduation rates, along with similar program satisfaction, to those without hypermobility. This finding suggests individuals with hypermobility can enroll, attend, and graduate multidisciplinary pain rehabilitation without significant modifications. Although PRP supports evidence-based interventions for patients with chronic pain, it does not provide specific materials on hypermobility across modalities. For example, while physical therapy, occupational therapy, and dietary support are tailored to hypermobility, psychology groups include all patients and thus are not designed to address the specific needs of people with hypermobility. Pending patient preference and demand, it may be possible to enroll hypermobile-only cohorts that provide comprehensive tailored programming specific to their needs.

## Data Availability

The raw data supporting the conclusions of this article will be made available by the authors, without undue reservation.
