# Supplementary material for: Symptomatic joint hypermobility is not a barrier to attendance, graduation, or satisfaction for adults participating in a multidisciplinary pain rehabilitation program
Source: Front Pain Res (Lausanne). 2025 Mar 20;6:1472160. doi: 10.3389/fpain.2025.1472160 (PMC11967196; doi:10.3389/fpain.2025.1472160)
Supplement: Supplementary file 1 [file Table1.docx]

**Supplemental Table 1: Breakdown of Diagnostic Code Frequency Among Patients in Hypermobile Group**

| **ICD-10 Codes** | **Count** |
| --- | --- |
| Q79.6 & M35.7 & Q79.9 | 6 |
| Q79.6 & M35.7 | 18 |
| Q79.6 & Q79.9 | 6 |
| Q79.6 | 24 |
| M35.7 | 4 |
| Q79.9 | 2 |
| Total | 60 |
| Abbreviations: Q79.6 = Ehlers-Danlos syndrome, M35.7 = Hypermobility syndrome, Q79.9 = Congenital malformation of connective tissue | |
